# Complete chloroplast genome of the tiny marine diatom *Nanofrustulum shiloi* (Bacillariophyta) from the Adriatic Sea

**DOI:** 10.1080/23802359.2019.1673245

**Published:** 2019-10-04

**Authors:** Chunlian Li, Romain Gastineau, Monique Turmel, Andrzej Witkowski, Christian Otis, Ana Car, Claude Lemieux

**Affiliations:** a Ecological Institute, South China Normal University, Guangzhou, Guangdong, China;; b Institute of Marine and Environmental Sciences, University of Szczecin, Szczecin, Poland;; c Département de biochimie, de microbiologie et de bio-informatique, Institut de Biologie Intégrative et des Systèmes, Université Laval, Québec, Canada;; d Institute for Marine and Coastal Research, University of Dubrovnik, Dubrovnik, Croatia

**Keywords:** Plastid genome, group II intron, pennate araphid diatoms, phylogenomics, Staurosiraceae

## Abstract

We report the chloroplast genome sequence of *Nanofrustulum shiloi*, a tiny araphid pennate diatom collected from the Adriatic Sea. The 160,994-bp *N*. *shiloi* genome displays a quadripartite structure and its gene repertoire resembles those of other diatom chloroplast genomes. Besides the genes located in the inverted repeat, *psbY* is duplicated. A gene-poor region in the large single-copy region contains multiple ORFs sharing sequence similarities with plasmids and chloroplast ORFs found in other diatom species. The genome features a single intron, a group II intron in *petB*. Phylogenomic analysis identified *N. shiloi* at a basal position within the araphid 2 clade.

*Nanofrustulum shiloi* is a small benthic araphid diatom widely distributed in marine littorals (Li et al. 2018). In the Mediterranean Sea, this pennate diatom is well known for its toxicity against marine invertebrates (Ruocco et al. 2018). With a diameter ranging from 2.0 to 6.0 µm, *N. shiloi* has a potential for diatom-based nanotechnologies (Losic et al. 2010). No complete chloroplast genome has yet been published for the family Staurosiraceae to which this species belongs.

The *N*. *shiloi* strain (clone SZCZM404) we examined for DNA sequencing was collected in October 2013 from Lumbarda Beach on the Adriatic Sea (Croatia). The culture is available at the Culture Collection of the University of Szczecin (Poland), where permanent slides with cleaned frustules are also deposited. Total DNA was extracted following Doyle and Doyle (1990). Paired-end sequencing was conducted on the BGISEQ-500 platform by the Beijing Genomics Institute (Beijing, China). A total of 60 million 100-bp reads were obtained and assembled using SPAdes v. 3.10.1 (Bankevich et al. 2012). Contigs of chloroplast origin were identified by BLASTN and BLASTX searches. Gene annotations were performed using a custom-built suite of bioinformatics tools (Turmel et al. 2017).

The *N. shiloi* chloroplast genome maps as a circular molecule of 160,994 bp (GenBank: MN276191), with two copies of a large inverted repeat (IR) sequence (11,617 bp) separating the small (SSC, 44,989 bp) and large (LSC, 92,774 bp) single-copy regions. It encodes 128 proteins of known function, 3 rRNAs, 27 tRNAs, tmRNA (ssrA), and the signal recognition particle RNA (ffs), for a total of 160 gene products. These genes are also usually encoded in the chloroplast in other diatoms (Yu et al. 2018). Two identical copies of the *N*. *shiloi psbY* gene lie side by side but on different strands in the SSC region, a characteristic apparently specific to this species. Note that *psbY* is located in the IR in several diatoms (Galachyants et al. 2012; Ruck et al. 2014; Sabir et al. 2014). The large intergenic region between *psaJ* and *psaA* in the LSC region harbors two putative serine recombinase genes (*serC*), annotated as *orf100* and *orf221*, as well as other ORFs showing similarities with coding sequences present in chloroplast genomes and plasmids of a number of raphid pennates (Hildebrand et al. 1992; Brembu et al. 2014; Ruck et al. 2014) and dinotoms (Imanian et al. 2010). The *N*. *shiloi* chloroplast genome features a single intron, a group II intron encoding a putative reverse transcriptase/maturase. This intron resides within *petB* at the same location as a similar intron found in the raphid *Halamphora calidilacuna* (Hamsher et al. 2019).

Maximum-likelihood and Bayesian inference trees were inferred from 129 concatenated chloroplast-encoded proteins of 42 diatom taxa using RAxML v.8.2.3 (Stamatakis 2014) and PhyloBayes v4.1 (Lartillot et al. 2009), respectively, as described by Lemieux et al. (2014). The results unambiguously positioned *N. shiloi* as the first divergence within the araphid 2 clade, which is sister to all raphid pennates (Figure 1).

**Figure 1.**
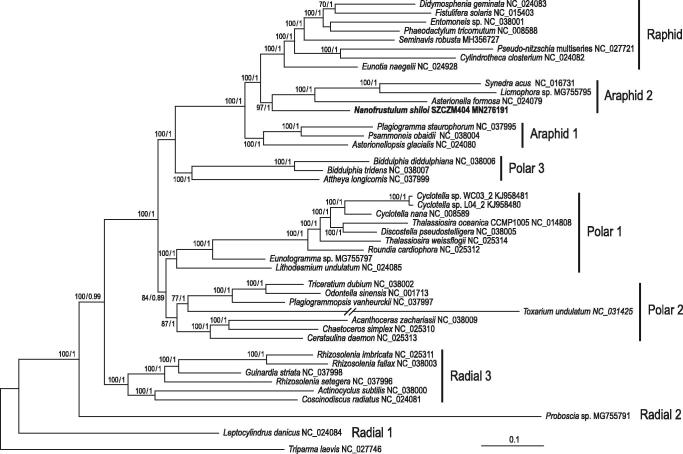
Phylogenetic analysis of 129 concatenated chloroplast-encoded proteins from 42 diatoms. The tree represents the best-scoring RAxML tree inferred under the GTR + Γ4 model. Support values are reported on the nodes, with bootstrap values for the RAxML analysis and the posterior probability values for the PhyloBayes CATGTR + Γ4 analysis shown from left to right, respectively. *Triparma laevis* (Bolidophyceae) was used to root the tree. The proteins selected for analysis are those conserved in most taxa. Clade labelling is identical to that in Yu et al. (2018). GenBank accession numbers for the chloroplast genomes of all taxa are provided. The scale bar denotes the estimated number of amino acid substitutions per site.
